# Transcaval approach for embolization of type II Endoleak following endovascular aortic aneurysm repair

**DOI:** 10.1186/s42155-018-0047-8

**Published:** 2019-01-09

**Authors:** Eddie Hyatt, Joseph N. McLaughlin, Hriday Shah, Sanjeeva P. Kalva

**Affiliations:** Division of Vascular and Interventional Radiology, Department of Radiology, University of Texas Southwestern Medical Center, 5323 Harry Hines Boulevard, Dallas, TX 75390 USA

**Keywords:** Abdominal aortic aneurysm, Endovascular aneurysm repair, Embolization, Transcaval embolization, Endoleak, Type II endoleak

## Abstract

Type II endoleak is a common complication following endovascular aortic aneurysm repair and can lead to an increased risk of aneurysmal expansion and rupture. The most frequently employed strategies to treat Type II endoleak involves catheterization of the branch vessels responsible for the endoleak or accessing the aneurysm sac through a percutaneous approach. An endovascular transcaval approach for embolization of the aneurysmal sac provides an alternate strategy with comparable success rates. This technique is advantageous when the endoleak is predominantly on the right side of the aneurysm sac and/or when a direct access to the aneurysm sac through a percutaneous approach is not feasible.

## Background

Endovascular abdominal aortic aneurysm repair (EVAR) is the standard of care for the treatment of most aneurysms greater than 5.5 cm diameter (Yang et al., 2016). Unfortunately, type 2 endoleak (T2E) is a common and often unavoidable complication resulting from incomplete exclusion of the aneurysm sac from the circulation via retrograde flow from branches of the abdominal aorta which can lead to aneurysm expansion and ultimately rupture.

Currently there is no consensus as to the optimal treatment strategy for T2E. The indication for treatment typically includes persistent endoleak (> 6 months) and continued sac expansion (> 0.5 cm) (Yamada et al., 2015; Ozdemir et al., 2013). Frequently used strategies include: 1) transarterial embolization of the feeding vessel accessed from the superior mesenteric or internal iliac artery collaterals; and 2) embolization via direct sac puncture from a translumbar (Ozdemir et al., 2013) or transabdominal (Zener et al., 2018) approach. The surgical options include ligation of the source vessels laparoscopically or open repair with feeding vessel ligation and aneurysmorraphy (Scali et al., 2013).

Transcaval embolization (TCE) provides an additional strategy with comparable success rates (Yang et al., 2016) while avoiding some limitations and reducing some risks inherent to other techniques, including technical failure due to inability to cannulate the feeding vessel, inadvertent injury to periaortic structures by traversing multiple tissue planes, prone patient positioning, and potential need for general anesthesia.

## Transcaval approach for Endoleak embolization

### Technique

Review of prior imaging is essential for optimal patient selection and procedure planning. The aneurysm sac ideally abuts the IVC and contains a minimal amount of wall calcification. From the bilateral groins or groin and neck an intravascular ultrasound (IVUS) probe is used to direct the tip of a vascular sheath against the inferior vena cava (IVC) wall near the site of the T2E identified on IVUS as patent cavities or inflow vessel tracts. The aneurysm sac is then punctured with a needle through the sheath using fluoroscopy and/or IVUS. Access of the endoleak cavity is confirmed by injection of dye. Wire and catheter are used to further select the endoleak cavity as needed. A microcatheter is eventually placed into the aneurysm sac and the endoleak cavity is embolized with coils, liquid embolics, or both**.**

### Case

**Fig. 1:**
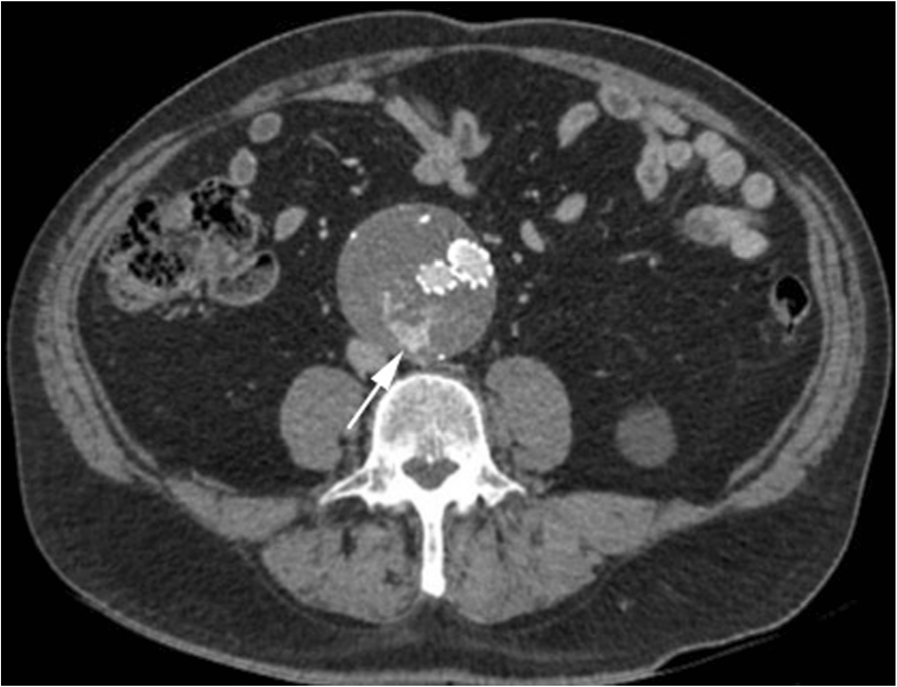
Axial computed tomography angiography (CTA) through the lower abdomen shows an abdominal aortic aneurysm with a T2E (white arrow) arising from an adjacent lumbar artery

A 75-year-old man underwent EVAR for a 6.1 cm abdominal aortic aneurysm. On follow-up CT angiography (CTA) imaging obtained 9 months later the aneurysm measured 6.8 cm and a T2E was seen arising from a lumbar artery (Fig. 1). Given the persistent T2E and continued enlargement of the aneurysm the decision to treat was made.

The procedure was performed under conscious sedation. The common femoral veins were accessed bilaterally with micropuncture sets (Vascular Solutions, Inc., Minneapolis, MN). The right femoral vein micropuncture sheath was exchanged for a Rosch-Uchida transjugular liver access set (Cook Medical, LLC, Bloomington, IN). The left femoral micropuncture sheath was exchange for a 9F vascular sheath through which an IVUS probe (Volcano Corporation, San Diego, CA) was advanced into the IVC.

**Fig. 2:**
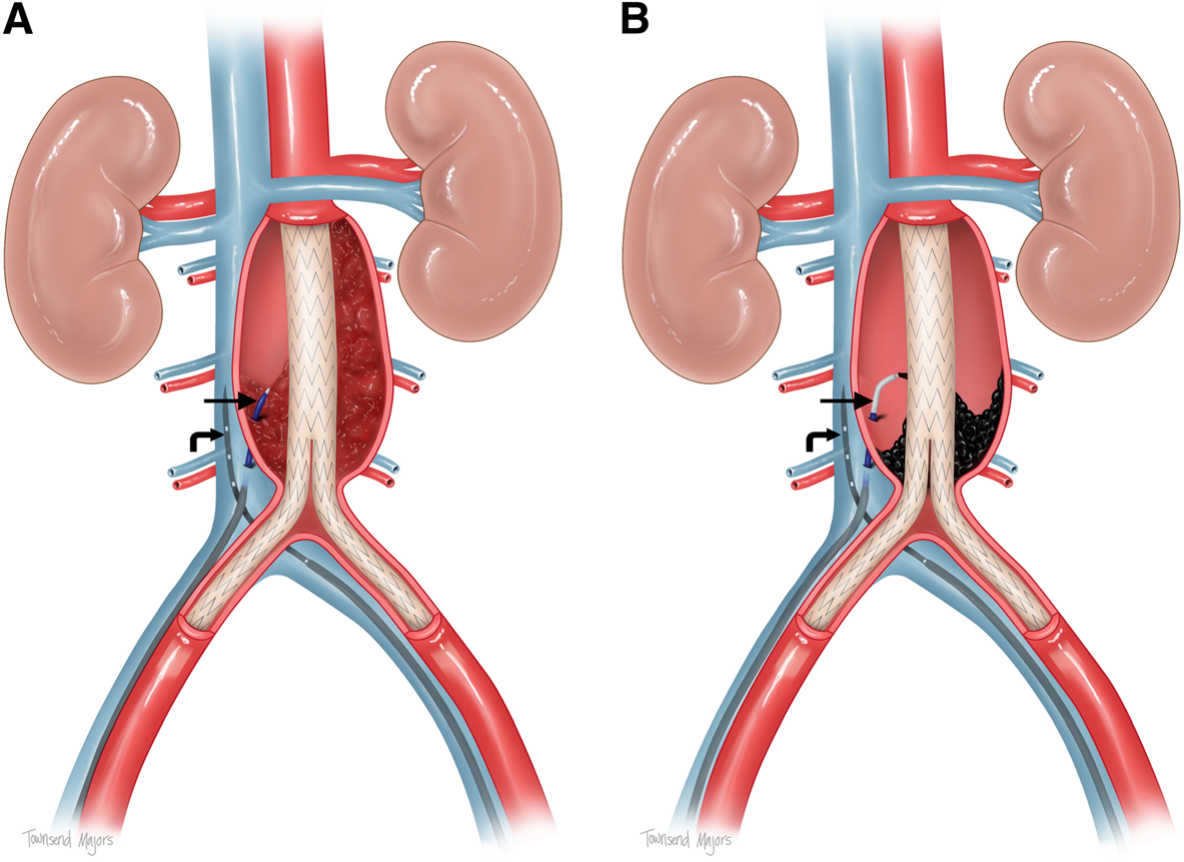
**a**. Rosch-Uchida liver access set (straight arrow) is used to access the aortic aneurysm sac. The aneurysm sac is accessed, near the site of the type II endoleak, from the inferior vena cava (IVC) under sonographic guidance with an intravascular ultrasound (IVUS) probe (curved arrow). **b**. The Rosch-Uchida needle was exchanged for an angle-tipped catheter and microcatheter (straight arrow). Embolic material is administered via the microcatheter. IVUS probe (curved arrow)

Under fluoroscopic and IVUS guidance the aneurysm sac was accessed near the endoleak with the Rosch-Uchida liver access set (Fig. 2). The inner needle was removed, and contrast was injected through the catheter confirming correct positioning within the aneurysm sac. This straight catheter was exchanged over the wire for a 5F angle tipped catheter (Terumo Medical Corporation, Somerset, NJ) which was used to select the endoleak cavity. A Progreat microcatheter/microwire set (Terumo Medical Corporation, Somerset, NJ) was advanced through the catheter into the aneurysm sac and position was confirmed with contrast injection.

**Fig. 3:**
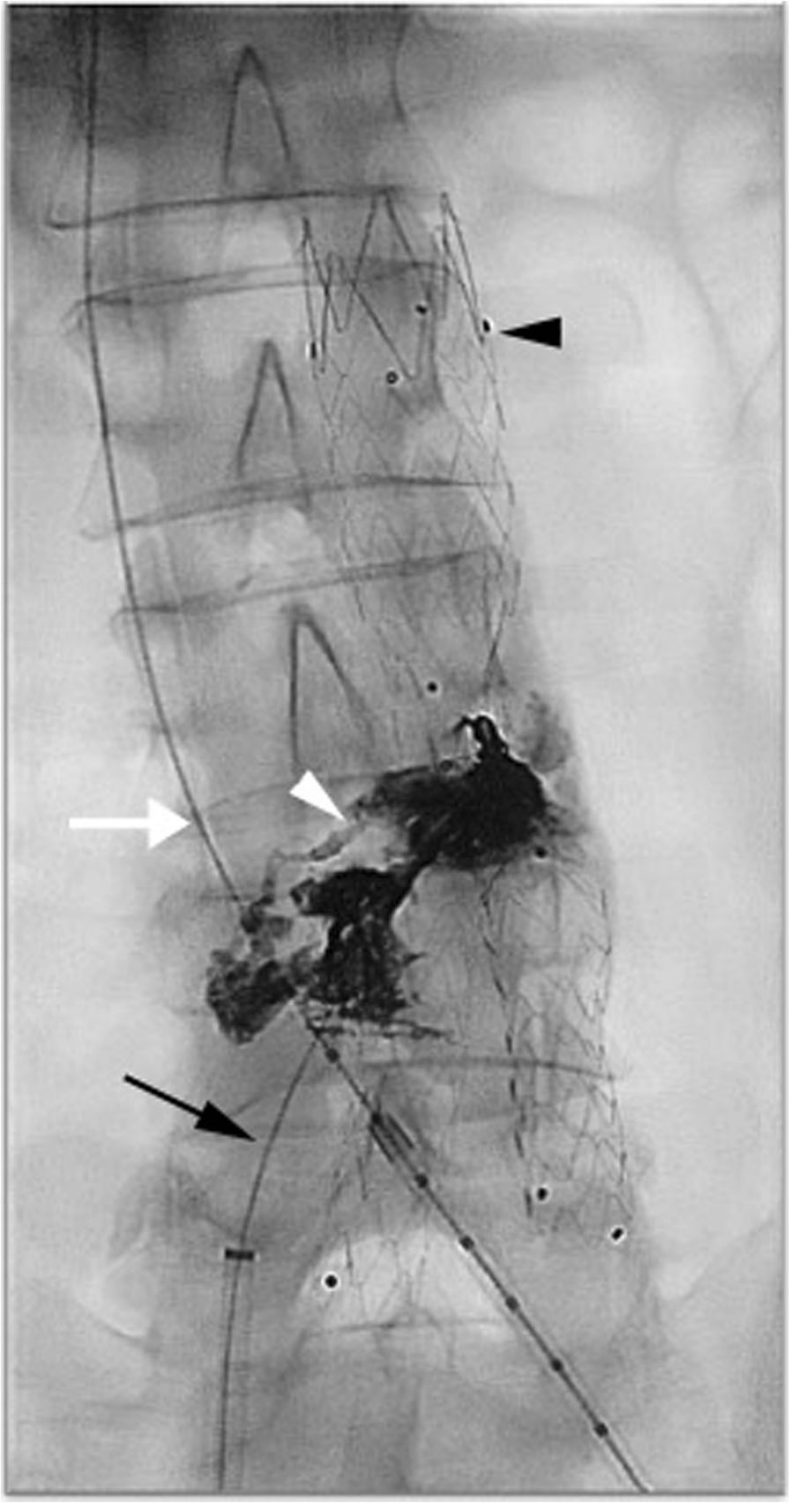
Fluoroscopic image obtained during the embolization procedure showing ethylene vinyl alcohol liquid embolic (Onyx®) administration via the microcatheter (black arrow). Guidewire and intravascular ultrasound (IVUS) probe (white arrow) within the IVC inserted through the left common iliac vein. Aortic stent graft (black arrowhead). Onyx® within the excluded aneurysm sac (white arrowhead)

**Fig. 4:**
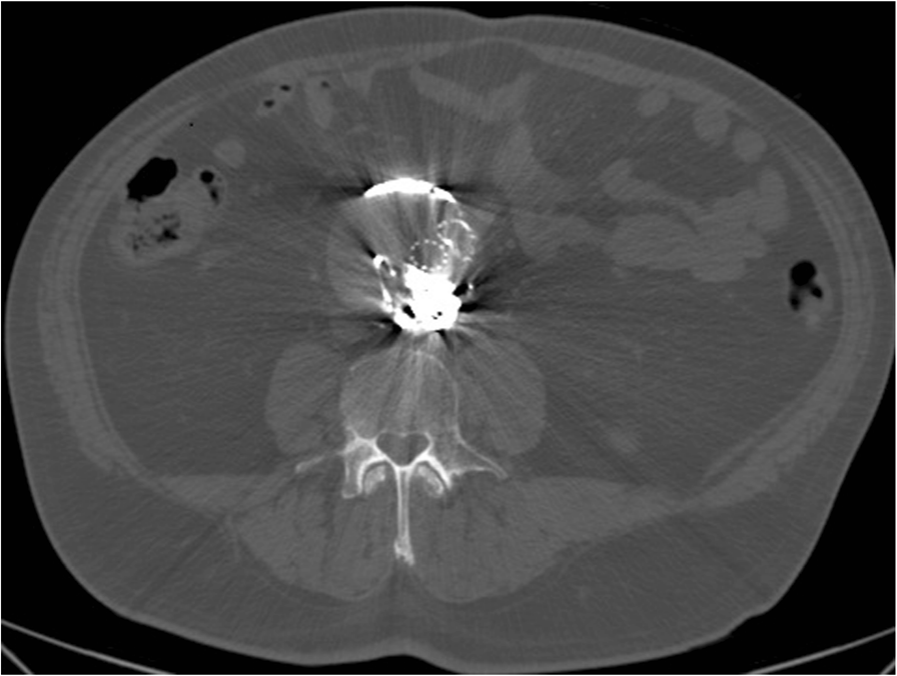
Post-embolization axial CTA through the lower abdomen at the same level as image in Fig. 1 shows embolic material filling the portion of the aneurysmal sac which previously demonstrated endoleak

Next, the microcatheter was flushed with 5% dextrose solution. Ethylene vinyl alcohol liquid embolic (Onyx®18) (Micro Therapeutics, Inc., Irvine, CA) was then administered through the microcatheter into the aneurysm sac, in the region of the T2E, under sonographic and fluoroscopic guidance (Figs. 2 and 3). The embolization was terminated once the endoleak was no longer visualized with IVUS. The catheters and IVUS probe were removed. Completion cavogram was performed through the femoral vein sheaths. Femoral vein sheaths were then removed, and hemostasis was achieved at the venotomy sites with manual compression. Total procedure time was 2.5 h. Follow up imaging (Fig. 4) demonstrated good radiographic results.

### Enhanced techniques

Real-time visualization of the access needle using “side-firing” IVUS probes is invaluable when performing TCE. First, it allows the endoleak cavity to be directly targeted. Targeted embolization of the endoleak cavity decreases the pressure within the aneurysm, increasing the likelihood of treatment success. Cessation of color Doppler flow on IVUS also determines the endpoint of embolization (Thakrar et al., 2013). (Alternatively, embolization can be stopped once intra-sac pressure stabilizes or has decreased by 50 mmHg (Gandini et al., 2014).) Appropriate endpoint determination decreases the risk of nontargeted embolization within the cava and/or pulmonary embolism. Use of IVUS also decreases the risk of creation of type III endoleak from unintentional puncture of the endograft, injury to periaortic/caval structures, aortocaval fistula formation, and retroperitoneal hemorrhage.

In thin patients, transabdominal ultrasound may work equally well. Post embolization, endoleak occlusion can also be confirmed in the angiosuite via real-time contrast enhanced US.

## Conclusion

T2E is a common, often unavoidable complication of EVAR which can lead to an increased risk of aneurysmal expansion and rupture. TCE is technically less demanding and faster (requiring less fluoroscopy time) approach to managing T2E. We believe TCE can potentially have a higher technical success rate and a decreased risk profile when compared with other more common catheterization techniques. Operators may consider TCE as a feasible first line alternative approach in the management of T2E.

## Abbreviations

AAA: Abdominal aortic aneurysm
CT: Computed tomography
CTA: Computed tomography angiography
EVAR: Endovascular abdominal aortic aneurysm repair
IVC: Inferior vena cava
IVUS: Intravascular ultrasound
T2E: Type II endoleak
TCE: Transcaval embolization
